# *De novo* Sequencing and Comparative Transcriptomics of Floral Development of the Distylous Species *Lithospermum multiflorum*

**DOI:** 10.3389/fpls.2016.01934

**Published:** 2016-12-23

**Authors:** James I. Cohen

**Affiliations:** Kettering UniversityFlint, MI, USA

**Keywords:** Boraginaceae, breeding system, distyly, herkogamy, heterostyly, *Lithospermum*, plant stress, transcriptome

## Abstract

Genes controlling the morphological, micromorphological, and physiological components of the breeding system distyly have been hypothesized, but many of the genes have not been investigated throughout development of the two floral morphs. To this end, the present study is an examination of comparative transcriptomes from three stages of development for the floral organs of the morphs of *Lithospermum multiflorum*. Transcriptomes of flowers of the two morphs, from various stages of development, were sequenced using an Illumina HiSeq 2000. The floral transcriptome of *L. multiflorum* was assembled, and differential gene expression (DE) was identified between morphs, throughout development. Additionally, Gene Ontology (GO) terms for DE genes were determined. Fewer genes were DE early in development compared to later in development, with more genes highly expressed in the gynoecium of the SS morph and the corolla and androecium of the LS morph. A reciprocal pattern was observed later in development, and many more genes were DE during this latter stage. During early development, DE genes appear to be involved in growth and floral development, and during later development, DE genes seem to affect physiological functions. Interestingly, many genes involved in response to stress were identified as DE between morphs.

## Introduction

**Figure 1:**
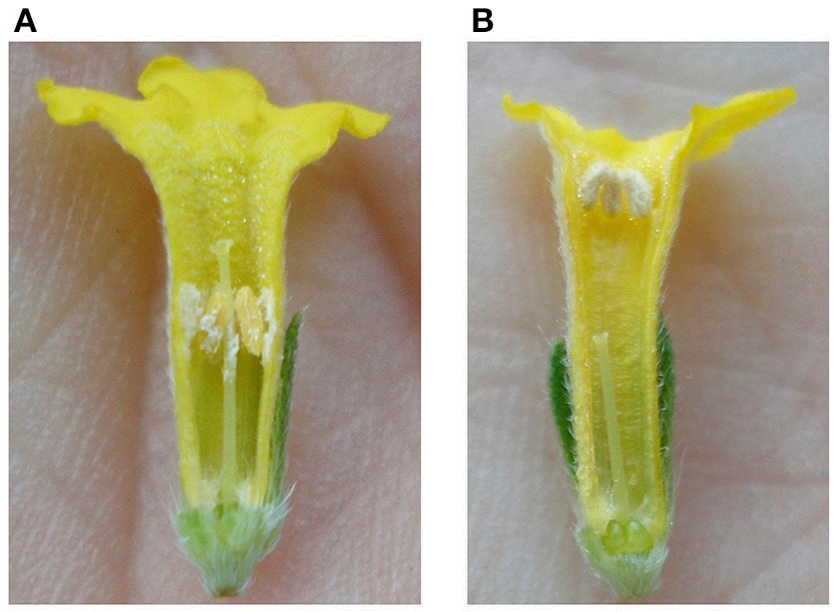
**Bisected flowers of the long-style (LS) (A)** and short-style (SS) **(B)** morphs of *Lithospermum multiflorum*.

Heterostyly is a complex and elegant breeding system characterized by the presence, in a species, of two or three floral morphs with sexual organs at distinct, yet fixed, heights (Barrett et al., 2000; Barrett and Shore, 2008). In heterostyly's simplest form, distyly, two floral morphs exist. The long-style (LS) morph has flowers with the stigma elevated above the anthers, and the short-style (SS) morph produces flowers in which the anthers are positioned above the stigma. The stigma in one morph is at the same height as the anthers in the other morph, a condition known as reciprocal herkogamy (Figure 1). In heterostyly, reciprocal herkogamy is usually accompanied by a self- and intramorph incompatibility (SI) mechanism that results in only sexual organs at the same height being compatible and able to produce fertile offspring. Researchers have demonstrated that together reciprocal herkogamy and SI allow for efficient pollen transfer among individuals of different morphs throughout populations, and this results in increased outbreeding and reduced inbreeding (Barrett, 1990; Ferrero et al., 2011; Zhou et al., 2015) [although see Björkman (1995) for an example of lack of efficient pollen transfer]. These functional and ecological aspects of heterostyly have been the subject of much research, dating back to Darwin's pivotal work on these components of the breeding system (1877), but the genetics of heterostyly, while investigated using numerous crossing studies (e.g., Ernst, 1928, 1933), still remain elusive (Fornoni and Domínguez, 2015; Gilmartin, 2015). One reason this is the case is that heterostyly is known from members of at least 28 families from across the angiosperms (Barrett and Shore, 2008; Cohen, 2010), but detailed data on the genetics of the breeding system is primarily limited to a small number of species (Gilmartin, 2015; Nowak et al., 2015). Indeed, much of the information on the genetics of heterostyly has been informed by studies on *Primula* L., and in this taxon, evidence suggests that the heterostylous condition is controlled by a supergene (*S*-locus), multiple genes that are tightly linked together, involved in the control of the breeding system (Barrett and Shore, 2008; Charlesworth, 2016). Outside of *Primula*, evidence for the presence of a supergene is weaker, primarily due to lack of data (Barrett and Shore, 2008; Cohen, 2010; Thompson and Jiggins, 2014). The present study employs de novo sequencing and comparative transcriptomics of three developmental stages of the flowers of the two morphs of the distylous species *Lithospermum multiflorum* Torr. ex A. Gray (Boraginaceae) in order to identify genes involved in the control of the heterostylous condition.

The genetics of heterostyly was initially characterized through crossing studies conducted during the late 1800s and early 1900s (Darwin, 1877; Ernst, 1928, 1933), and the outcomes of these studies resulted in the hypothesis of the three-gene *S*-locus (Lewis, 1954; Dowrick, 1956; Richards, 1997; Nowak et al., 2015). Recent studies have focused on uncovering the underlying genetics of a small number of heterostylous species, including *Linum* L., *Turnera* L., and *Primula* (McCubbin et al., 2006; Somers et al., 2008; Labonne et al., 2009; Ushijima et al., 2012, 2015). Many of the studies have recognized genes or proteins either linked to the *S*-locus or with morph-specific patterns of expression.

In *Linum*, Ushijima et al. (2012, 2015) identified a gene, *TSS1*, that was tightly linked to the *S*-locus and only segregated with SS individuals, while other genes unlinked to the *S*-locus, such as *LgSKS1*, which are also highly expressed in the SS morph, have post-transcriptional regulation. This latter finding suggests that the development of the two floral morphs is controlled by more than just the components of the *S*-locus. In *Turnera*, Somers et al. (2008) and Labonne et al. (2009) constructed a genetic map of the *S*-locus and identified three genes, *TkNACA, TkST1*, and *TsRETRO*, linked to it. Further studies on *Turnera*, using X-ray-generated mutants, allowed gene order to be postulated for the *S*-locus (Labonne et al., 2010).

Research on *Primula* has been more extensive (Gilmartin, 2015; Nowak et al., 2015; Huu et al., 2016), and recent work has resulted in the identification of multiple genes associated with the *S*-locus, such as *PVeGLO2*, a GLOBOSA homolog, and *CYP734A50*, both present in *Primula veris* L. *PVeGLO2*, which was identified via a draft genome of the species, is not expressed in the LS morph (Nowak et al., 2015). Similarly, *CYP734A50* is only expressed in the styles of the SS morph, and the loss of the expression of this gene results in the elongated styles of the LS morph (Huu et al., 2016). Using BAC contigs, Li et al. (2015) identified a one megabase (Mb) region that contains 82 genes linked to the *S*-locus, and this region appears to be located close to the centromere. That location may be one of the reasons that recombination among the genes in the *S*-locus occurs infrequently (Talbert and Henikoff, 2010). In a related species of *Primula, P. vulgaris* Huds., McCubbin et al. (2006) utilized subtractive cDNA libraries of morph-specific floral tissue to identify differentially expressed (DE) genes between the flowers of the two morphs. These researchers identified 11 DE genes involved in a variety of different processes, including stress response, cell wall development, and RNA-binding; however, none of these DE genes were linked to the *S*-locus. This provides evidence that multiple genes are involved in downstream processes of morph-specific floral development in heterostylous species. Additionally, genes and proteins that differ between morphs have been studied in other heterostylous taxa, such as *Fagopyrum* Mill. (Fesenko and Fesenko, 2006) and *Averrhoa* L. (Wong et al., 1994; Fesenko and Fesenko, 2006), but to a lesser extent.

While identification of the *S*-locus is important in understanding heterostyly, at least in some species, the breeding system provides an excellent opportunity to investigate genes involved in floral organ height and length. In most species, identification of these genes would require large quantitative trait loci (QTL) studies involving examination of the range of morphological variation and associated molecular markers [e.g., over 6500 plants observed in a QTL study on flower size of *Erythranthe* Spach (formerly *Mimulus* L.) (Kelly and Monica, 2011)]. Given the distinct differences in floral organ heights and lengths in heterostylous species, these species do not succumb to these issues. Consequently, examining floral transcriptomes of heterostylous species, at various stages of development, allows for the identification of genes involved in controlling the sizes of flowers and flower organs. It is possible to compare patterns of gene expression of flowers of the morphs of heterostylous species because the flowers of the morphs develop following the same genetic program, with the only exceptions hypothesized to be those resulting in the morph-specific differences (Cohen, 2010). Genes that are differentially expressed between the morphs should be those that are involved in heterostyly. While the *S*-locus, at least in *Primula*, may be the genomic region ultimately controlling the heterostylous condition, other genes from throughout the genome may be involved in development of the breeding system (McCubbin et al., 2006), with identification of the *S*-locus only providing limited data.

*L. multiflorum* is a distylous species (Figure 1) common in the intermountain west in North America (Cronquist et al., 1984). The species is a member of Boraginaceae, the family that has the largest number of independent origins of heterostyly, and this type of breeding system has evolved multiple times in *Lithospermum* L. (Cohen, 2011). Cohen et al. (2012) demonstrated that there are differences in the development of the flowers within the genus, and data concerning SI and floral morphology and development based on studies by Philipp and Schou (1981), Casper (1985), Li and Johnston (2010), Ferrero et al. (2012), and Cohen (2014) suggest that heterostyly evolved following distinct patterns in different tribes of the family. Heterostyly arose independently in *L. mulitflorum*, and the species is sister to *Lithospermum macromeria*. Cohen (2011), a species with floral morphology quite different from *L. multiflorum* (i.e., long, green to white corollas and exserted anthers and stigmas). Consequently, *L. multiflorum* is located at an intriguing phylogenetic position for an investigation of patterns of morph-specific flower gene expression. To this end, the present study is an examination of the comparative transcriptomics of (1) the corolla and androecium and (2) the gynoecium of the two morphs of *L. multiflorum* at three stages of development in order to recognize genes that are differentially expressed between the morphs, and, therefore, regulating floral morphology in this species.

## Materials and methods

### Plant material, RNA extraction, and transcriptome sequencing

Flowers of the two morphs of *L. multiflorum* were collected from three populations in and around Flagstaff, Arizona, USA during June 2011 and 2012. In order to capture appropriate developmental stages, multiple flowers of different sizes were taken from plants, and after quickly removing some sepals, flowers were immediately placed in RNAlater (Applied Biosystems, Foster City, CA, USA), and subsequently stored either at 4°C overnight or placed on dry ice. All specimens were then stored indefinitely at −20°C. Voucher specimens for the populations were made (Cohen 353, 354, 356, and 398), and specimens were deposited at MICH.

**Figure 2:**
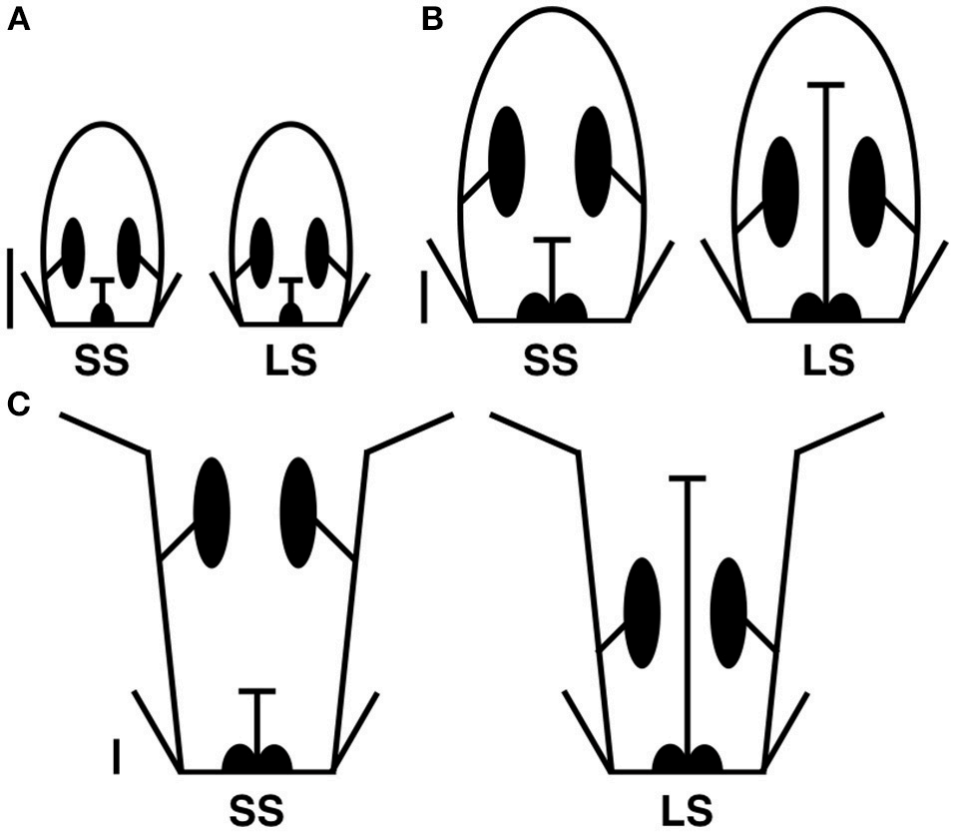
**Representations of stages of development for short-style (SS) and long-style (LS) morphs, (A)** early, **(B)** mid, **(C)** late. Approximate 1 mm scale bars left of each pair of flowers.

Flowers were divided into three stages—early, middle, and late—based on the developmental patterns identified by Cohen et al. (2012) (Figure 2). Flowers in the early stage are up to 4 mm in length, which is before morph-specific differences are apparent in the flowers. Flowers in the middle stage are 4–9 mm in length, which is after morph-specific floral differences are observed in heights of anthers and stigmas. Flowers in the late stage are greater than 9 mm in length, and these flowers range from near anthesis to anthesis, with morph-specific differences well-defined. For each morph at each stage, three extractions were made for the organ group of the corolla, androecium, and gynoecium, and two extractions were conducted for the organ group of the corolla and androecium, resulting in a total of 30 extractions (Table S1). Flowers for RNA extraction were arbitrarily selected from either 2011 or 2012, and given the similar environmental conditions around Flagstaff, AZ during June of the 2 years (https://www.wunderground.com/), it would appear that different collection times would have minimal impact on variation in patterns of gene expression.

RNA was extracted from one or two RNAlater-preserved flowers using a modification of the Li-Cl method of Jaakola et al. (2001). Right before RNA isolation, the remaining sepals and pedicel were removed. Before the extraction buffer was added, flowers were homogenized using the method outlined in Alexander et al. (2007). The primary modification from Jaakola et al. (2001) involved the extraction buffer, which in the present study included 2% CTAB, 2% PVP (Mol WT 40,000), 0.5% SDS, 100 mM Tris- HCl (pH 8.0), 25 mM EDTA, 2.0 M NaCl, and 0.5 g/L spermidine, with 2% β-mercaptoethanol and 1.5 mg/mL Proteinase K added just before use. After RNA was isolated, the quantity, 260/280, and 260/230 ratios were determined using a Nanodrop ND-1000 spectrophotometer (Thermofisher Scientific, Waltham, MA, USA). RNA that was at least a total of 1 μg and with 260/280 and 260/230 > 1.8 was treated with the TURBO DNA-*free*™ Kit (Applied Biosystems) following the manufacturer's protocol. Subsequently, isolations were sent to the Duke University Genome Sequencing & Analysis Core Resource, where the quality of the RNA was determined via an Agilent 2200 TapeStation (Agilent Technologies, Santa Clara, CA, USA), and then prepared for transcriptome sequencing, on an Illumina HiSeq 2000, using the TruSeq RNA Kit. One extraction from each stage from each group of organs (total of 12) was sequenced using 100 base pair (bp) paired-end (PE) reads across three lanes, and the remaining extractions (18) were sequenced using 100 bp single-reads (SR) across five lanes. Afterwards, adapters were trimmed and low quality and short reads were removed at the Duke University Genome Sequencing & Analysis Core Resource. Sequence data are deposited at the Sequence Read Archive (SRP093214, BioProject PRJNA353131).

### Transcriptome assembly and mapping

The transcriptome of *L. multiflorum* was assembled, at Duke University, based on the 12 PE transcriptomes from the three developmental stages of the two morphs. Using the ~389 million high-quality pairs of reads sequenced from the 12 RNA isolations, the floral transcriptome of the species was assembled using Trinity v 1 (Grabherr et al., 2011). The transcriptome was annotated and gene ontology terms identified (GO terms) using the Integrative Services for Genomic Analysis (http://isga.cgb.indiana.edu). In order to test for DE genes between the morphs and organs at various stages of development, the protocol of Van Verk et al. (2013) was followed. FASTQ files were uploaded to the Galaxy server (usegalaxy.org), and five base pairs were trimmed from the ends of each read, followed by filtering the reads to remove those with low quality scores (reads <90% of the bp with a Phred quality score of ≥20). After filtering, reads from each RNA isolation were mapped to the isoforms of the Trinity-assembled transcriptome. Using Bowtie (Galaxy tool version 1.1.2) (Langmead et al., 2009), reads were mapped, with the appropriate settings for SR and PE employed, using the default (lenient) settings as well as with a stringent criterion (i.e., -a,-best, -strata, -m 1, -n 3, -l 30, -e 288, -y off, -k 1, -maxbts 800). The SAM files from the Bowtie runs were analyzed using eXpress (Roberts and Pachter, 2013), with the Galaxy server, to determine the number of reads that mapped to each isoform. This was undertaken using three different methods: Default settings and two more thorough analyses, involving two additional rounds of each of the batch and online expectation-maximization (EM) algorithm, a mean fragment length either of 90 bp for all reads or 90 bp for SR and 190 bp for PE, and a standard deviation of one. Total counts and effective counts, which are counts adjusted for fragment and target length biases, were recorded (Roberts and Pachter, 2013).

### Differential gene expression and gene ontology

The total number of reads mapped to each isoform for each putative gene were compiled in order to determine the number of reads mapped to each putative gene. To test for similarity between transcriptomes from the same developmental stage and morph, Pearson correlations were calculated in Microsoft Excel. For each putative gene and isoform, both the total and effective counts from eXpress from the two Bowtie mapping strategies were analyzed in edgeR v.3.0.0 (Robinson et al., 2010) to test for differential expression (DE) of transcripts among floral organs at early, middle, and late stages of floral development for the corolla and androecium and for the gynoecium. Putative DE genes were identified based on a *P*-value ≤ 0.05 and a false discovery rate (FDR), using the Benjamini-Hochberg adjustment, of ≤0.1 (Anders et al., 2013). To investigate overlap of DE genes among morphs, organs, and stages of development, venn diagrams were constructed with VennDiagram (Chen and Boutros, 2011). Hierarchical clustering of DE genes was conducted across the three developmental stages, for the two organ groups, using NMF (Gaujoux and Seoighe, 2010).

After the DE putative genes and isoforms were identified for each organ or group of organs at the three developmental stages, genes were annotated using Blast2GO (Conesa et al., 2005) running blastx on the nr database at NCBI, and GO terms were determined, using Blast2GO, with InterProScan (Zdobnov and Apweiler, 2001) and by mapping genes with the Gene Ontology Database (GO Consortium, 2004). With GOstats 2.34.0 (Falcon and Gentleman, 2007), GO terms for the DE genes for each organ or group of organs for each developmental stage were compared to those for all genes in the floral transcriptome in which GO terms were identified. GO terms for Biological Processes, Cellular Components, and Molecular Function were identified that were over- and underrepresented, based on a *P*-value ≤ 0.5. The hyperGTest in GOstats was run with and without the conditional algorithm. For these analyses, because of the small number of DE genes and the similarity to those during early development, the mid-stage DE genes were included with those from the early stage of development.

## Results

### Transcriptome sequencing, assembly, and mapping

A total of 30 transcriptomes were sequenced for flowers at the three stages of development for the two morphs of *L. multiflorum*. For the paired-end (PE) reads, the total number of reads for a transcriptome ranged from 58,474,582 to 134,651,292 100-bp reads. For the single reads (SR), the total number of reads for a transcriptome ranged from 38,244,776 to 78,986,321 100-bp reads. The assembled floral transcriptome of *L. multiflorum* included 97,603 components (putative genes) and 265,144 isoforms (putative gene variants). The minimum length of a putative gene was 201 bp, and the maximum assembled length was 16,847 bp. For the genes, the average length was 690 bp, with an N50 of 1201 bp, and for the isoforms, the average length was 1144 bp, with an N50 of 2029 bp. Statistics for the assembled transciptome are provided in Table 1. Approximately 2–8% of the total reads were removed via the filtering and trimming steps. Using the default (lenient) and more stringent Bowtie settings, ca. 70–80 and 40% of the reads mapped to the assembled transcriptome, respectively. Data on the number of reads are presented in Table S1.

**Table 1:** **Statistics on assembled transcriptome of ***Lithospermum multiflorum*****.

**Statistics**	**Genes**	**Isoforms**
Minimum length	201	201
Maximum length	16,847	16,847
Mean length	691	1144
Standard deviation	844	1142
Median length	361	661
N50 length	1201	2029
Number of assembled reads >1 kb	16,724	103,530
Number of isotigs in N50	14,109	48,516
Number of bases in all isotigs	67,417,306	303,313,357
Number of bases in isotigs ≥1 kb	36,575,211	232,659,690
GC content of isotigs	38.53%	38.55%

### Differential gene expression and gene ontology

The Pearson correlations for the components and isoforms were greater than 0.85 for the corolla and androecium and for corolla, androecium, and gynoecium at early and late stages of development, suggesting high correlation among the transcriptomes. For the middle stages of development, some of the transcriptomes had high Pearson correlations (>0.85), particularly for the transcriptomes of the LS corolla, androecium, and gynoecium; however, most of the others had weaker associations, with values ranging from 0.4 to 0.57 (Table S2).

The two different mapping criteria in Bowtie (lenient and stringent), in conjunction with the six different methods for determining the number of reads mapped, in eXpress [total and effective counts for each of (1) no specified fragment length, (2) a mean fragment length of 90 bp for all reads, and (3) a mean fragment length of 90 bp for SR and 190 bp for PE], resulted in 12 manners in which the number of differentially expressed (DE) genes were calculated (Table 2). Of the 12 different manners in which DE genes were calculated, the lenient Bowtie analyses tended to result in more DE genes identified, with edgeR, compared to the more stringent Bowtie analyses. Additionally, the use of effective counts, rather than total counts, generally resulted in fewer DE genes. Specifying the length of the mean fragments at either 90 bp for each read or 190 bp for PE reads and 90 bp for SR, did not seem to influence the number of DE genes as much as the mapping criteria and the use of total or effective counts, with the former resulting in fewer DE genes compared to the latter (Table 2).

**Table 2:** **Number of differentially expressed genes from analyses of developmental stages and organs, see text for explanation of different mapping and differential expression (DE) methods**.

**Developmental stage**	**Bowtie lenient, 90 + 190 bp length, effective counts**	**Bowtie lenient, 90 + 190 bp length, total counts**	**Bowtie stringent, 90 + 190 bp length, effective counts**	**Bowtie stringent, 90 + 190 bp length, total counts**	**Bowtie lenient, no length, effective counts**	**Bowtie lenient, 90 bp length, effective counts**	**Bowtie lenient, 90 bp length, total counts**	**Bowtie lenient, no length, total counts**	**Bowtie stringent, no length, effective counts**	**Bowtie stringent, no length, total counts**	**s Bowtie stringent, 90 bp length, effective counts**	**Bowtie stringent, 90 bp length, total counts**	**Total unique genes**
Early corolla	53	59	0	0	47	59	53	59	13	13	0	0	68
Mid corolla	1	0	0	0	0	0	1	1	0	1	0	0	1
Late corolla	260	1344	0	0	239	1344	262	1344	172	1089	0	256	1444
Early gynoecium	13	33	1	11	13	12	33	76	2	20	1	11	111
Mid gynoecium	7	17	1	4	7	17	2	17	1	3	1	4	22
Late gynoecium	1127	1593	309	812	1023	1125	1593	1310	906	1523	311	812	3135
Total	1408	2987	311	827	1282	2498	1891	2748	1081	2636	313	1083	4713

**Figure 3:**
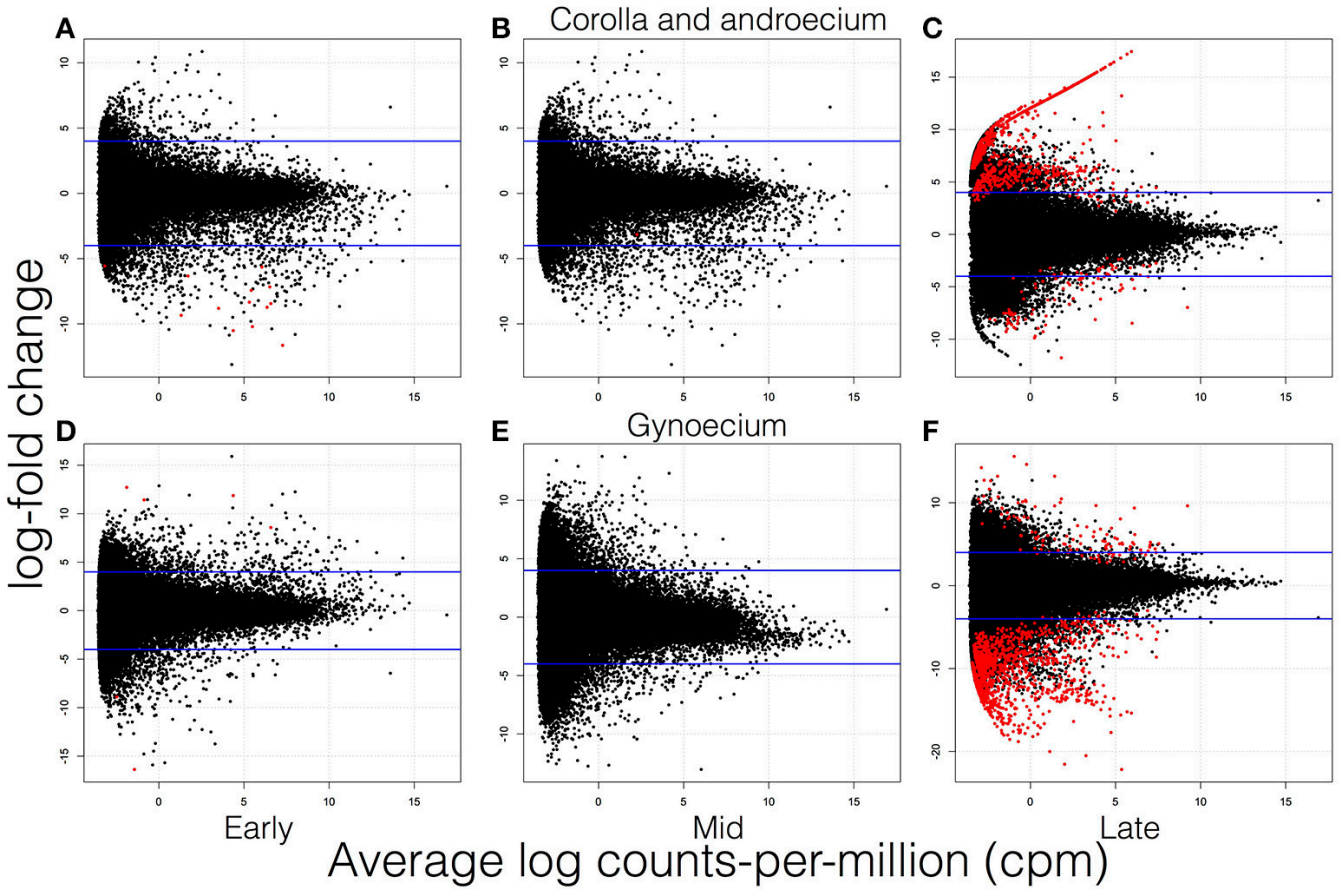
**Volcano plots of differentially expressed genes of floral transcriptomes of ***Lithospermum multiflorum*** for organs throughout developmental stages**. **(A–C)** are for corolla and androecium, and **(D–F)** are for gynoecium. **(A)** and **(D)** are early development, **(B)** and **(E)** are mid-development, and **(C)** and **(F)** are late development. Red dots are genes significantly differentially expressed between morphs, and blue lines represent four log-fold changes. Positive values represent genes more highly expressed in long-style morph, and negative values signify genes more highly expressed in short-style morph.

**Figure 4:**
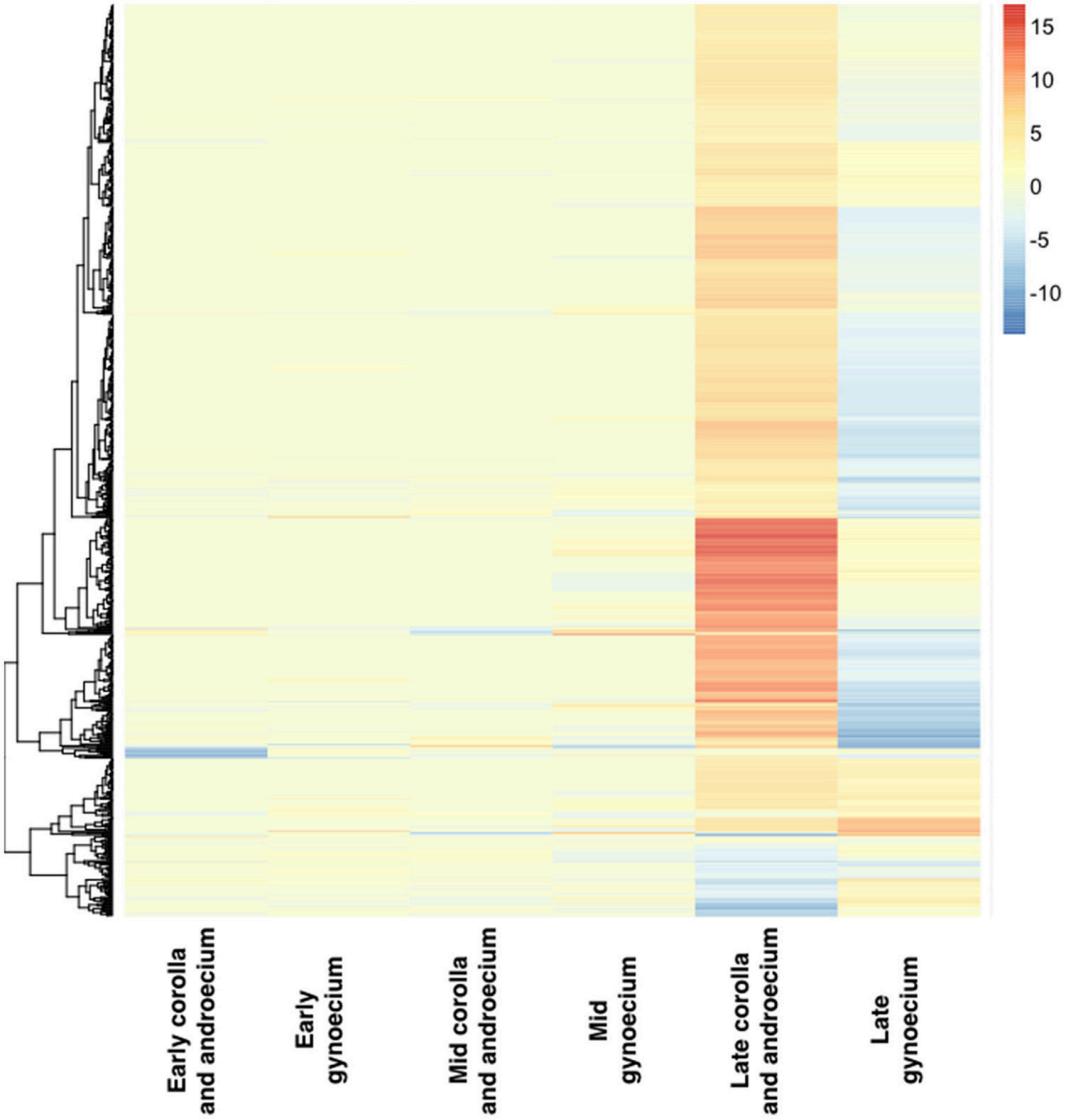
**Hierarchical clustering and heat map of organs at three stages of development based on log-fold changes in expression of total counts from analysis using default Bowtie and eXpress settings, red signifies greater expression in long-style (LS) morph, and blue denotes greater expression in short-style (SS) morph**. Scale refers to log-fold changes in expression.

In the corolla and androecium, more DE genes early in development were more highly expressed in the short-style (SS) morph (although this was a small number), while a greater number of DE genes were more highly expressed in the long-style (LS) morph during late development (Figures 3, 4). The opposite pattern was observed in the gynoecium, with more highly expressed DE genes in the LS morph during early development (again, a small number), and many more DE genes more highly expressed in the SS morph during late development of the gynoecium (Figures 3, 4). Indeed, the greatest number of DE genes between morphs was observed in the gynoecium during the late stage of growth. Only a small number of DE genes was identified during the mid-stage of development both for the corolla and androecium and for the gynoecium, with the smallest number of DE genes across stages and organs identified in the corolla and androecium of the mid-stage of development (Table 2). The DE genes that were identified in the greatest number of analyses, for each stage and organ, are presented in Table 3. This number ranged from 4 to 12 depending on the stage and organ combination. The majority of these genes were successfully annotated using Blast2GO, and most exhibit greater than six log-fold changes, in expression, between morphs.

**Table 3:** **Statistics and putative identity of differentially expressed (DE) genes of developmental stages and organs most frequently identified from across analytical methods, positive and negative values in log-fold change are greater expression in long-style (LS) and short-style (SS) morphs, respectively**.

**Early corolla DE genes**	**Putative gene**	**Number of analyses gene DE**	**Log-fold change**	**Log counts-per-million**	**Likelihood ratio**	***P*-value**	**False discovery rate**
comp113449	Myosin family protein with dil domain	8	–7.2464	5.3816	24.1378	^**^	0.0219
comp94365	Glutamine dumper	8	–10.5977	3.4677	24.8753	^**^	0.0235
comp107848	Continuous vascular ring family protein	8	–11.9474	4.6562	23.0457	^**^	0.0262
comp112590	Hypothetical protein MIMGU_mgv11b0241222mg, partial	7	–12.5420	6.1440	23.0341	^**^	0.0262
comp111181	Pleckstrin homology domain-containing family A member 8	8	–5.7277	5.1564	21.7499	^**^	0.0312
comp117098	Lysine histidine transporter 1	8	–8.3750	6.7616	21.8987	^**^	0.0312
comp102592	lactoylglutathione lyase family protein	8	–7.5394	4.5240	20.8535	^**^	0.0329
comp106343	n/a	8	–8.8766	2.5666	21.0607	^**^	0.0329
comp98284	Hypothetical protein MIMGU_mgv1a016237mg	8	–9.0030	5.4421	21.0653	^**^	0.0329
comp115443	Hypothetical protein CICLE_v10002660mg	7	–6.4899	4.0016	20.6916	^**^	0.0329
comp89953	PREDICTED: uncharacterized protein LOC101259110	7	–9.9241	4.4621	19.5456	^**^	0.0458
comp104913	n/a	8	–9.2570	0.2433	18.5503	^**^	0.0589
comp75402	Heavy metal-associated isoprenylated plant protein 26-like	7	–9.5382	3.4367	16.9332	^**^	0.0844
**MID COROLLA DE GENES**							
comp90112	Carotenoid cleavage dioxygenase chloroplastic-like	4	–8.1503	1.3315	25.4450	^**^	0.0444
**LATE COROLLA DE GENES**							
comp1117673	n/a	10	14.6336	2.0912	16.8029	^**^	0.0143
comp4162239	Sugar transport protein 10-like	10	12.9209	0.1315	16.8089	^**^	0.0143
comp43701	Beta-galactosidase 13-like	10	13.0543	0.2890	16.7540	^**^	0.0144
comp44582	n/a	10	12.6192	–0.2367	16.7332	^**^	0.0144
comp79612	Cysteine proteinase rd21a-like	10	12.8943	0.0973	16.7229	^**^	0.0144
comp2645921	Endoglucanase 16-like	10	13.3491	0.6361	16.6791	^**^	0.0145
comp88548	Pathogenesis-related maize seed protein	10	16.3996	3.9490	16.6826	^**^	0.0145
comp1603779	Ribosome associated membrane protein ramp4	10	12.7303	–0.0992	16.5179	^**^	0.0149
comp2540996	n/a	10	12.4408	–0.4503	16.5224	^**^	0.0149
comp2683611	Pectinesterase pectinesterase inhibitor 28-like	10	13.2777	0.5510	16.5344	^**^	0.0149
comp65255	Pollen allergen ole e 6-like	10	14.8540	2.3301	16.5087	^**^	0.0149
comp95123	L-ascorbate oxidase homolog	10	16.3423	3.8915	16.4475	0.0001	0.0154
comp1701545	n/a	10	14.2074	1.6195	16.3711	0.0001	0.0158
comp3100697	n/a	10	13.0010	0.2217	16.3370	0.0001	0.0159
comp2190644	Olee1-like protein	10	13.7895	1.1464	15.9304	0.0001	0.0173
comp1767292	Pectinesterase pectinesterase inhibitor 28-like	10	13.3356	0.6230	15.8839	0.0001	0.0175
comp1986942	n/a	10	13.0874	0.3276	15.6488	0.0001	0.0187
comp2830467	Pollen-specific protein sf3-like	10	14.1237	1.5271	15.3355	0.0001	0.0204
comp107951	Wat1-related protein at3g28050-like	10	9.8738	–1.2539	15.0876	0.0001	0.0215
comp2384086	Arabinogalactan protein 14	10	13.2555	0.5273	14.8195	0.0001	0.0233
**EARLY GYNOECIUM DE GENES**							
comp127830	241k polyprotein	7	–6.4500	13.5994	29.1451	^**^	0.0022
comp88015	Cp protein	7	–16.3710	–1.4412	28.0797	^**^	0.0028
comp106811	Beta-galactosidase 13-like	5	3.8066	14.1328	27.7009	^**^	0.0028
comp108439	Probable n-acetyltransferase hls1-like	5	11.0583	2.6327	26.2899	^**^	0.0036
comp118914	Probable ccr4-associated factor 1 homolog 7-like	5	0.9771	14.9701	24.4516	^**^	0.0062
comp47121	n/a	6	12.7101	–1.8928	24.6451	^**^	0.0096
comp102300	Aldose 1-epimerase-like	6	0.7863	14.7067	23.9491	^**^	0.0121
comp94365	Glutamine dumper	9	12.4072	4.7351	25.9591	^**^	0.0170
comp119774	Phospholipase a1- chloroplastic-like	5	–4.2863	2.8680	21.5807	^**^	0.0184
comp100745	Rapid alkalinization factor 23-like	5	3.7056	13.9542	21.1962	^**^	0.0405
comp1174059	n/a	6	11.4198	–0.8758	20.9731	^**^	0.0413
comp123276	Subtilase family protein	5	5.3276	14.5848	19.2794	^**^	0.0501
comp107848	Continuous vascular ring family protein	5	11.4145	4.6562	19.0432	^**^	0.0542
comp14260	n/a	6	6.8712	–2.5756	19.2161	^**^	0.0760
comp112590	Hypothetical protein MIMGU_mgv11b0241222mg, partial	6	12.0240	7.2663	18.3478	^**^	0.0945
comp50910	PREDICTED: uncharacterized protein LOC101253901	6	15.9010	4.2792	18.4226	^**^	0.0945
comp75402	Heavy metal-associated isoprenylated plant protein 26-like	5	11.3739	3.4367	17.2721	^**^	0.0958
**MID GYNOECIUM DE GENES**							
comp127830	241k polyprotein	8	–4.2982	12.4529	39.6674	^**^	^**^
comp118914	Probable ccr4-associated factor 1 homolog 7-like	6	–2.7357	14.9701	25.1782	^**^	0.0085
comp106811	Beta-galactosidase 13-like	5	–2.3629	13.3177	23.8908	^**^	0.0142
comp129234	n/a	5	15.0468	3.4172	23.1366	^**^	0.0184
comp118394	n/a	5	6.1655	4.0752	21.9946	^**^	0.0243
**LATE GYNOECIUM DE GENES**							
comp115916	Polygalacturonase at1g48100-like	12	–10.9082	4.6929	78.1199	^**^	^**^
comp127830	241k polyprotein	12	–1.9522	12.4529	40.1043	^**^	^**^
comp102478	Non-specific lipid-transfer protein at5g64080-like	12	9.2553	3.3612	33.8880	^**^	^**^
comp110356	Cysteine-rich repeat secretory protein 60-like	12	–10.8093	2.0296	30.3242	^**^	0.0001
comp104697	Aspartic proteinase-like protein 1-like	12	–13.8727	2.1802	30.1851	^**^	0.0001
comp102300	Aldose 1-epimerase-like	12	2.8449	13.8822	27.0702	^**^	0.0003
comp116177	Serine threonine-protein kinase pbs1-like	12	7.0065	1.5660	26.4948	^**^	0.0004
comp107662	n/a	12	–13.1629	0.4624	21.7722	^**^	0.0023
comp101506	Laccase diphenol oxidase family protein	12	–13.2946	–1.5837	19.4333	^**^	0.0049
comp124850	Glutaredoxin family protein	12	–4.6547	2.7362	17.0746	^**^	0.0107
comp16116	Agc kinase	12	–14.2467	–2.1013	15.6194	0.0001	0.0168
comp103048	Geraniol 8-hydroxylase-like	12	–9.7069	–1.2283	15.2436	0.0001	0.0191
comp126173	Protein-tyrosine kinase 2-beta-like	12	–5.6988	3.7806	13.1116	0.0003	0.0389
comp59609	Cytochrome p450 716b2-like	12	11.2034	–1.1369	12.9591	0.0003	0.0407
comp84994	n/a	12	11.2034	–1.1369	12.9591	0.0003	0.0407
comp93273	Early nodulin 55-2	12	13.0025	–1.9506	12.9543	0.0003	0.0407
comp95648	Sulfate transporter-like	12	–4.7264	2.2436	11.7373	0.0006	0.0623
comp110461	Nicotianamine synthase-like	12	–5.5001	1.1028	11.6528	0.0006	0.0637
comp121779	Mitochondrial glycoprotein family protein	12	–5.9671	1.4003	11.4604	0.0007	0.0678
comp97467	n/a	12	–9.8769	0.9120	10.5722	0.0011	0.0918
comp1218480	n/a	12	–8.5381	–3.0767	10.4282	0.0012	0.0962

** *Denotes P < 0.0001*.

**Figure 5:**
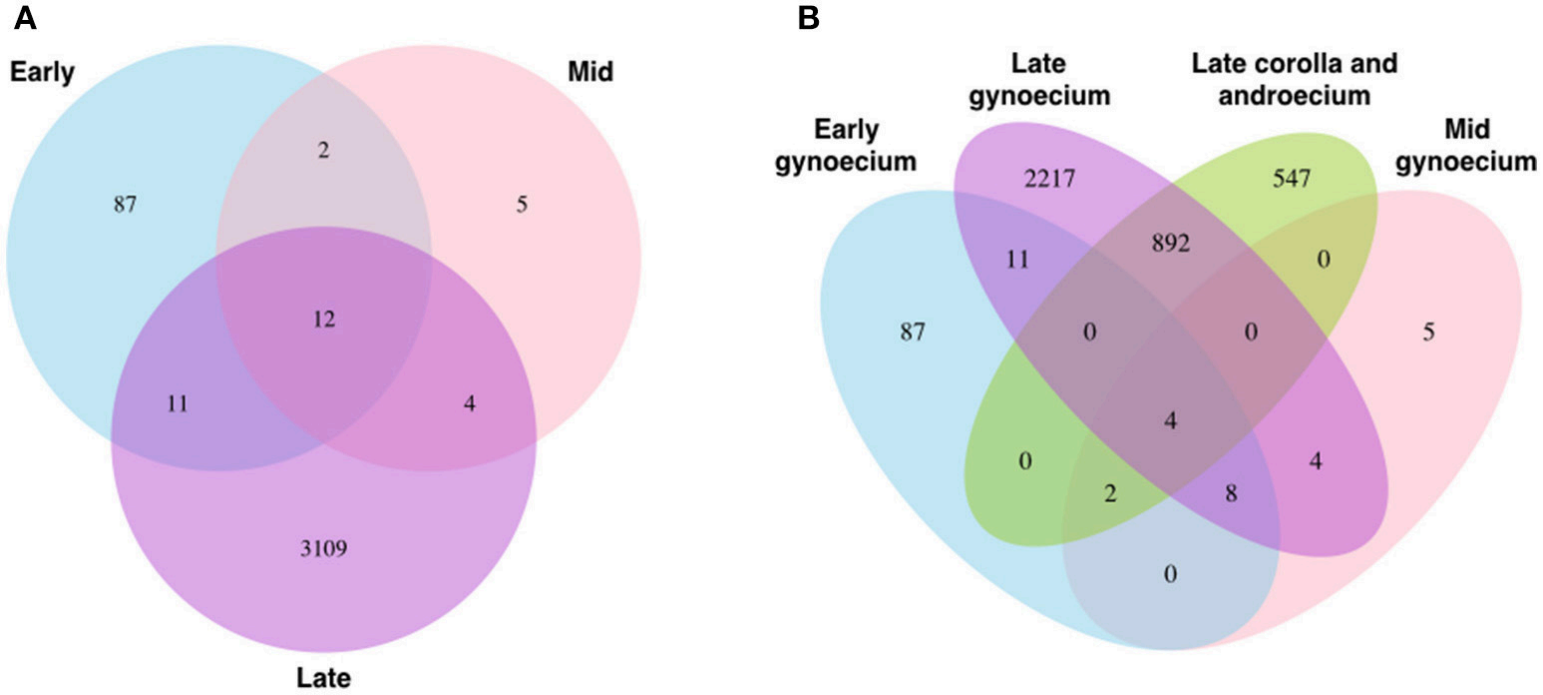
**Venn diagrams of patterns of differential gene expression (DE) of (A)** gynoecium throughout development and **(B)** three stages of gynoecial development and late-stage corolla and androecium.

No genes expressed in the corolla and androecium overlap in patterns of expression among the developmental stages. In contrast, 12 genes are DE in the gynoecium across all three developmental stages, with smaller numbers overlapping between the early and mid-stage of development (two) and the mid- and late stage (four) (Figure 5). Interestingly, 11 genes are DE in both the early and late stage of the gynoecium, but not in the mid-stage of development (Figure 5). The vast majority of genes DE during one stage are not DE at other stages (Figures 4, 5). However, 892 DE genes are common at the late stage of development for the corolla and androecium and the gynoecium, although the patterns of expression may differ among these genes (Figures 4, 5). Analyses of hierarchical clustering also illustrate that a large number of genes that are DE during the late stage are not DE during the earlier stages (Figure 4).

The pluralities of the Gene Ontology (GO) terms were identified from *Glycine max* (L.) Merr., *Vitis vinifera* L., *Populus trichocarpa* Torr. & A. Gray ex Hook., and *Citrus sinensis* Osbeck, although GO terms identified from species throughout the angiosperms were represented in the annotation. The top-hit species were primarily members of Lamiidae, including *Coffea canephora* Pierre ex A. Froehner, *Erythranthe guttata* (DC.) G. L. Nesom, *Solanum tuberosum* L., and *Solanum lycopersicum* L., as well as *V. vinifera*. A total of 65,535 GO terms were identified for the floral transcriptome of *L. multiflorum*. GO terms were overrepresented during all stages of development, and only in the early and mid-stage of the development of the corolla and androecium were no GO terms underrepresented (Table 4). During early and mid-development, most of the GO terms that differed in frequency between DE genes and the transcriptome as a whole were overrepresented in the DE gene set, whereas during late development, a greater number were underrepresented in the DE gene set (Table 4).

**Table 4:** **Number of Gene Ontology (GO) terms over- and underrepresented during developmental stages of the floral organs**.

**GO term type**	**Early corolla and androecium**	**Early gynoecium**	**Late corolla and andreocium**	**Late gynoecium**	**Total**
Biological processes overrepresented	165	38	30	41	210
Biological processes under-represented	0	23	199	281	223
Cellular components overrepresented	21	5	8	11	36
Cellular components under-represented	0	1	45	49	57
Molecular function overrepresented	45	10	8	15	61
Molecular function under-represented	0	12	107	150	159

A summary of the most over- and underrepresented GO terms is presented in Table 5. Many more genes are DE during late floral development compared to during early and mid-floral development (Figures 4, 5), regardless of the type of analysis (Table 2). Early in development, gene ontology (GO) terms that are overrepresented, for Biological Processes (BP), Cellular Components (CC), and Molecular Function (MF), appear to be involved in growth. For example, GO terms associated with membrane fusion, actin cytoskeleton organization, cell tip, cell pole, site of polarized growth, and actin binding are overrepresented early in development in the corolla and androecium of the SS morph (Tables 5, 6). During late development, more genes are DE compared to early development, and the genes that are DE differ between the two stages. Later in development, many DE genes appear to be involved in physiological functions, which can include self- and intramorph-incompatiblity and response to stress. Indeed, it is worth noting that response to stress is the only GO term overrepresented throughout the developmental stages in all investigated organs when the conditional algorithm of GOstats was not employed, and this GO term was one of only three BP GO terms overrepresented when the condition algorithm was used (Table 6). As with the patterns of DE genes in general, more of those involved in response to stress are expressed later in development than earlier in development. While specific GO terms related to distinct responses to stress, such as those related to the regulation of particular hormones, were not found to be over- or underrepresented, some genes that respond to hormones known to be involved in response to stress (e.g., ethylene and abscisic acid) were DE during various stages of development (Table 7). During late development, the BP and MF GO terms overrepresented, in both the corolla and androecium and the gynoecium, may be involved in SI, including cell wall organization, cytoskeletal protein, and others (Tables 5, 6). Additionally, overrepresented CC GO terms tend to be either extracellular or toward the periphery of the cell, which is not the case for overrepresented CC GO terms early in development, which are more internal to the cell or involved in growth, such as an overrepresentation of plant-type vacuole membrane and intracellular organelle during early development but not latter development (Tables 5, 6). Genes putatively involved in these functions and locations are overexpressed in the corolla and androecium of the LS morph and in the gynoecium of the SS morph.

**Table 5:** **Five most significant Gene Ontology (GO) terms over- and underrepresented during developmental stages of the floral organs**.

**Biological processes overrepresented**	**Early corolla and androecium**	***P*-value**	**Early gynoecium**	***P*-value**	**Late corolla and androecium**	***P*-value**	**Late gynoecium**	***P*-value**
	Lipid storage	^**^	Response to biotic stimulus	0.0017	Cell wall organization or biogenesis	^**^	Cell wall organization or biogenesis	^**^
	Membrane fusion	^**^	Stem vascular tissue pattern formation	0.0036	Growth	^**^	Membrane organization	^**^
	Ammonium transport	0.0001	N-terminal protein myristoylation	0.0036	Cell differentiation	^**^	Lipid metabolic process	^**^
	Actin cytoskeleton organization	0.0003	Amino acid import	0.0036	Membrane organization	^**^	Growth	^**^
	Sulfur amino acid metabolic process	0.0003	Basic amino acid transport	0.0036	Carbohydrate metabolic process	^**^	Reproduction	^**^
**BIOLOGICAL PROCESSES UNDER-REPRESENTED**								
	N/A		Cellular biosynthetic process	0.0003	Oxidation-reduction process	^**^	Organonitrogen compound biosynthetic process	^**^
			Single-organism cellular process	0.0025	Response to inorganic substance	^**^	Oxidation-reduction process	^**^
			Single-organism metabolic process	0.0031	Metal ion transport	^**^	Response to inorganic substance	^**^
			Organonitrogen compound biosynthetic process	0.0084	Nucleobase-containing compound biosynthetic process	^**^	Small molecule biosynthetic process	^**^
			Phosphorus metabolic process	0.0085	Cellular macromolecule biosynthetic process	^**^	Regulation of macromolecule metabolic process	^**^
**CELLULAR COMPONENTS OVERREPRESENTED**								
	Pollen tube tip	0.0031	Mitochondrion	0.0002	Extracellular region	^**^	Extracellular region	^**^
	Site of polarized growth	0.0031	Intracellular organelle	0.0138	Cell periphery	^**^	Cell periphery	^**^
	Cell tip	0.0031	Nuclear lumen	0.0419	Cell wall	^**^	Cell wall	^**^
	Central vacuole	0.0031	Membrane-bounded organelle	0.0423	Plasma membrane	^**^	Thylakoid	^**^
	6-phosphofructokinase complex	0.0031	Plant-type vacuole membrane	0.0476	Golgi apparatus	^**^	Golgi apparatus	^**^
**CELLULAR COMPONENTS UNDER-REPRESENTED**								
	N/A		Protein complex	0.0426	Intracellular organelle part	^**^	Integral component of membrane	^**^
					Integral component of membrane	^**^	Intracellular organelle part	^**^
					Membrane part	^**^	Chloroplast	^**^
					Chromosome	^**^	Membrane part	^**^
					Chloroplast	^**^	Integral component of endoplasmic reticulum membrane	^**^
**MOLECULAR FUNCTION OVERREPRESENTED**								
	Actin binding	0.0003	Glutathione dehydrogenase (ascorbate) activity	0.0036	Glutathione dehydrogenase (ascorbate) activity	0.0036	Enzyme regulator activity	^**^
	Transferase activity, transferring acyl groups	0.0021	Carbohydrate binding	0.0054	Carbohydrate binding	0.0054	Peptidase activity	^**^
	Carbohydrate kinase activity	0.0026	Enzyme regulator activity	0.0057	Enzyme regulator activity	0.0057	Nucleic acid binding transcription factor activity	^**^
	6-phosphofructokinase activity	0.0027	Acidic amino acid transmembrane transporter activity	0.0071	Acidic amino acid transmembrane transporter activity	0.0071	Cytoskeletal protein binding	^**^
	UDP-glucuronate decarboxylase activity	0.0027	Neutral amino acid transmembrane transporter activity	0.0071	Neutral amino acid transmembrane transporter activity	0.0071	Oxidoreductase activity	^**^
**MOLECULAR FUNCTION UNDER-REPRESENTED**								
	N/A		Anion binding	^**^	Organic cyclic compound binding	^**^	Organic cyclic compound binding	^**^
			carbohydrate derivative binding	0.0002	heterocyclic compound binding	^**^	heterocyclic compound binding	^**^
			Purine ribonucleotide binding	0.0002	Anion binding	^**^	Anion binding	^**^
			Ribonucleoside binding	0.0002	Nucleotide binding	^**^	Nucleotide binding	^**^
			Purine nucleoside binding	0.0002	Carbohydrate derivative binding	^**^	Carbohydrate derivative binding	^**^

** *Denotes P < 0.0001*.

**Table 6:** **Most common Gene Ontology (GO) terms over- and underrepresented during floral development**.

**Gene ontology terms**	**Early corolla and androecium *P*-value**	**Early gynoecium *P*-value**	**Late corolla and androecium *P*-value**	**Late gynoecium *P*-value**
**BIOLOGICAL PROCESSES OVERREPRESENTED**				
Intracellular transport	0.0046		0.0101	^**^
Response to stress		0.0262	0.0015	0.0001
Symbiosis, encompassing mutualism through parasitism		0.0214	0.0423	0.0031
**BIOLOGICAL PROCESSES UNDERREPRESENTED**				
Regulation of transcription, DNA-templated		0.0446	^**^	^**^
Cellular amino acid metabolic process		0.0337	0.0001	^**^
Regulation of biosynthetic process		0.0403	^**^	^**^
Phosphorylation		0.0392	^**^	^**^
Regulation of nucleobase-containing compound metabolic process		0.0419	^**^	^**^
Nucleobase-containing compound biosynthetic process		0.0223	^**^	^**^
Oxoacid metabolic process		0.0153	^**^	^**^
Cellular biosynthetic process		0.0003	^**^	^**^
Small molecule biosynthetic process		0.0330	^**^	^**^
Single organism reproductive process		0.0326	^**^	^**^
Carboxylic acid biosynthetic process		0.0405	^**^	^**^
Nucleic acid-templated transcription		0.0383	^**^	^**^
Organonitrogen compound biosynthetic process		0.0084	^**^	^**^
Regulation of RNA biosynthetic process		0.0446	^**^	^**^
**CELLULAR COMPONENTS OVERREPRESENTED**				
Extracellular region			^**^	^**^
Cell wall			^**^	^**^
Peroxisome			0.0013	0.0043
Golgi apparatus			^**^	^**^
Microtubule organizing center			0.0197	0.0070
Plasma membrane			^**^	0.0001
Plant-type vacuole membrane	0.0431	0.0476		
Intracellular organelle	0.0363	0.0138		
Cell periphery			^**^	^**^
**CELLULAR COMPONENTS UNDERREPRESENTED**				
Ubiquitin ligase complex		^**^	^**^	^**^
Chromatin		^**^	^**^	^**^
Nucleosome		^**^	^**^	^**^
Cell		0.0003	^**^	0.0003
Nucleus		0.0215	0.0005	0.0220
Chromosome		^**^	^**^	^**^
Cytoplasm		^**^	^**^	^**^
Mitochondrial envelope		0.0001	^**^	0.0001
Mitochondrial inner membrane		0.0002	0.0001	0.0002
Vacuolar membrane		^**^	^**^	^**^
Endoplasmic reticulum membrane		^**^	^**^	^**^
Spindle		0.0004	^**^	0.0004
Cytosol		0.0025	^**^	0.0025
Microtubule		^**^	^**^	^**^
Glycerol-3-phosphate dehydrogenase complex		^**^	^**^	^**^
**MOLECULAR FUNCTION OVERREPRESENTED**				
Enzyme regulator activity		0.0057	0.0057	^**^
**MOLECULAR FUNCTION UNDER-REPRESENTED**				
Purine nucleoside binding	0.0002	^**^	^**^	0.0002
ATP binding	0.0005	^**^	^**^	0.0005
Phosphotransferase activity, alcohol group as acceptor	0.0401	^**^	0.0342	0.0744
Active transmembrane transporter activity	0.0434	0.0028	^**^	0.0462
Adenyl nucleotide binding	0.0004	^**^	^**^	0.0004
Ribonucleoside binding	0.0002	^**^	^**^	0.0002
Purine ribonucleotide binding	0.0002	^**^	^**^	0.0002
Anion binding	^**^	^**^	^**^	^**^
Carbohydrate derivative binding	0.0002	^**^	^**^	0.0002

*Lack of numbers in a cell represents lack of significant over- or underrepresentation*.

** *Denotes P < 0.0001*.

**Table 7:** **Statistics and putative identity of differentially expressed (DE) genes identified as involved in response to hormones ethylene or abscisic acid; positive and negative values in log-fold change are greater expression in long-style (LS) and short-style (SS) morphs, respectively**.

**Organ and component**	**Putative gene**	**Log-fold change**	***P*-value**	**False discovery rate**
**EARLY COROLLA DE GENES**				
comp109661	ca+2-binding ef hand family protein	−7.8602	^**^	0.0875
**EARLY GYNOECIUM DE GENES**				
comp111457	Ethylene-responsive transcription factor 1b	4.7616	0.0001	0.0830
**MID COROLLA DE GENES**				
comp90112	Carotenoid cleavage dioxygenase chloroplastic-like	−8.1503	^**^	0.0444
**LATE COROLLA DE GENES**				
comp113274	Ethylene-responsive transcription factor erf034-like	−3.6415	0.0001	0.0229
comp115033	Ethylene-responsive transcription factor erf034-like	−9.6793	0.0002	0.0284
comp125113	Abscisic acid receptor pyr1-like	−2.8589	0.4133	0.9142
**LATE GYNOECIUM DE GENES**				
comp109390	Nodulin-related protein	5.3946	0.0001	0.0145
comp97404	Ethylene-responsive transcription factor erf012-like	−13.3995	^**^	0.0128
comp108406	Ethylene-responsive transcription factor erf017-like	12.0045	^**^	^**^
comp110611	Ethylene-responsive transcription factor win1-like	−3.6710	0.0012	0.0923
comp111802	Ethylene receptor	−3.6710	0.0012	0.0923
comp113114	Ap2-like ethylene-responsive transcription factor bbm2-like	−10.6153	0.0005	0.0512
comp114072	Ethylene-responsive transcription factor rap2-11-like	4.8431	0.0001	0.0254
comp116180	Ethylene-responsive transcription factor rap2-4	−4.2944	0.0005	0.0531
comp122880	Ethylene-responsive transcription factor rap2-7-like	5.0128	^**^	0.0026

** *Denotes P < 0.0001*.

## Discussion

### Floral transcriptome of *Lithospermum multiforum*

The present study demonstrates the utility of transcriptomics to identify differentially expressed genes in flowers, throughout development, of a heterostylous species. The transcriptome of *L. multiforum* includes 97,603 putative genes and 265,144 isoforms of these genes. In comparison to other floral transcriptomes of heterostylous species, this is a much larger number of genes. For example, *Fagopyrum, Primula*, and each of two species of *Eichhornia* Kunth express ca. 25,400, 55,000, and 20,000—24,000 genes, respectively (Logacheva et al., 2011; Ness et al., 2011; Zhang et al., 2013). Additionally, the transcriptome of *L. multiflorum* is greater than that of the only other species of Boraginaceae, *Echium wildpretii* H. Pearson ex. Hook.f., with an assembled transcriptome, which includes ca. 58,500 genes and 69,500 transcripts (White et al., 2016). The number of putative genes and transcripts of *E. wildpretii* is greater than that of the other aforementioned species as well, suggesting the possibility that the species of Boraginaceae may have large transcriptomes.

The large number of putative genes of the *L. multiflorum* transcriptome could be due to a variety of different factors, including the complexity of the transcriptome of the species, the inclusion of multiple stages of development in the construction of the transcriptome, paralogs due to an ancient polyploidy event in the genus (*N* = 14, Ward and Spellenberg, 1988), or technical issues, such as the assembly of incomplete contigs due to solely using transcriptome, not genome, data. Additionally, the flowers used in the present study were collected at different stages of development from wild populations, which could result in the expression of genes that may not have been expressed under more controlled conditions. Therefore, particular genes, such as those involved in stress, may not be expressed if conditions are more stable for the plants. However, it should be noted that all of the plants used in the present study were growing under similar conditions (including between years), even if these were in the native environment of the species, which should result in a negligible impact on DE solely due to environmental factors.

### Bowtie and eXpress criteria

The present study employed different mapping criteria and parameters for quantifying reads mapped. Unsurprisingly, the more stringent mapping criteria employed in Bowtie (Langmead et al., 2009) resulted in fewer reads mapped, and subsequently fewer DE genes identified using edgeR (Robinson et al., 2010). In general, the use of effective counts, in eXpress (Roberts and Pachter, 2013), resulted in the identification of fewer DE genes, as did the addition of length information. It is notable that using multiple criteria can result in different numbers of DE genes identified. Indeed, the most conservative methods employed found ca. 10% of the DE genes identified with the more lenient approaches. By utilizing different criteria, it was possible to identify a suite of genes DE across multiple analyses, which may be a useful approach for determining genes to target for future research, such as real-time PCR and *in-situ* hybridizations, to better identify patterns of expression among related homostylous and hetersotylous species of *Lithospermum* and Boraginaceae.

### Differential expression of genes throughout development

During early development, genes overexpressed in the corolla and androecium of the SS morph have been demonstrated to be involved in cell division and actin dynamism, and this includes particular genes that are DE, such as myosin family protein with dil domain and pleckstrin homology domain-containing family A member 8 (Lehman et al., 1996; Berg et al., 2000). In contrast, genes overexpressed in the developing gynoecium of the LS morph, such as n-acyltransferase hls-1 like, have been shown to control cell growth via auxin regulation (Table 3) (Lehman et al., 1996; Berg et al., 2000; Peremyslov et al., 2010). Together, this pattern of expression suggests that different genes are involved in the elongation and growth of various organs in the developing flower bud. Later in development, DE genes and GO terms have been demonstrated to be involved in pollen-specific responses, reproduction, and cell signaling, all of which may play a role in SI. During this stage of development, DE genes include pollen allergen ole e 6-like protein, ole e 1-like protein, pollen-specific protein sf3-like, and protein-tyrosine kinase 2-beta-like (Table 3), while overrepresented CC GO terms comprise extracellular region, cell wall, plasma membrane (Table 6) (Baltz et al., 1992a,b; Hubbard and Till, 2000; Staiger and Franklin-Tong, 2003; Jiménez-López et al., 2011; Lopez-Casado et al., 2012). This pattern of expression demonstrates a shift in the function of DE genes between early and late development.

The small number of DE genes involved in growth expressed early in development, along with the larger number involved in physiological processes expressed later in development, provide evidence that the morphological components of heterostyly (i.e., herkogamy) are controlled by a smaller number of genes than those involved in the physiological aspects of the breeding system (i.e., SI). This suggests that fewer changes in patterns of expression would need to develop in order to result in shifts in herkogamy compared to changes in SI, which is consistent with the model for the evolutionary development proposed by Lloyd and Webb (1992) and supported by phylogenetic and developmental data in *Lithospermum* (Cohen, 2011; Cohen et al., 2012), hypothesizing that reciprocal herkogamy developed prior to SI in the genus. Furthermore, some heterostylous species in Boraginaceae (e.g., *Oreocarya flava* A. Nelson) have SI that is not fully established (Casper, 1985), so the morphological components would have arisen without the origin of SI.

Along with the disparity in the number of DE genes at different stages of development, there is an asymmetry present during late development in the pattern of expression of DE genes in floral organs. In the corolla and androecium, the majority of DE genes are overexpressed in the long-style (LS) morph, while in the gynoecium, the situation is reversed, with the majority of the DE genes overexpressed in the short-style (SS) morph (Figures 3–5). While this pattern is reversed during early development, it is certainly not as pronounced owing to the much smaller number of DE genes during earlier stages of development (Figures 3–5). This late-development asymmetry is notable because it has been demonstrated that SI does not necessarily act in the same manner in each morph in heterostylous species (Schou and Philipp, 1984). This has not been studied in *L. multiflorum*, but given the different patterns of expression in the floral organs of the two morphs, this may be the case, with the pollen from the LS morph interacting with the gynoecium of SS morph in a different way than that of the pollen of SS morph with the gynoecium of LS morph.

The mid-stage of development differs from the early and late stages of development in two manners—a much smaller number of DE genes identified and low intramorph Pearson correlations. Fewer than 25 DE genes were identified for the mid-stage of development, with fewer than 20 for any of the individual analyses (Figure 4). This number is less than one third of the DE genes during early development and far smaller than the DE genes during late development (Table 3). This could be due to the mid-stage not being reflective of a distinct biological stage of development. There may be an early stage that establishes morphological differences, followed by a later stage in which SI develops. Therefore, the use of a mid-stage of development, in the present study, may be artificial and include flowers at both the early and late stages of development, which could result in the lower Pearson correlations, as opposed to the high Pearson correlations for floral transcriptomes within the respective early and late stages of development. This variation may also result in fewer DE genes due to differences among the biological replicates of the flowers at the mid-stage.

A limited number of studies have used comparative transcriptomics to identify genes involved in herkogamy and heterostyly, and it is notable that some of the genes identified as DE in other taxa are also DE in *L. multiflorum*. Ness et al. (2011) compared the floral transcriptomes of two species of *Eichhornia* that include selfing and outcrossing representatives. One hundred and forty-seven genes were upregulated in the flowers of the selfing individuals studied, with 10 genes identified as being involved in pollination or flower development. Ness et al. (2011) extracted RNA from flowers at various stages of development, but the RNA was pooled for sequencing. Of these 10 DE genes, two auxin response factors were identified, and this was also the case for early development in the flowers of *L. multiflorum*. During late development, six genes similar to those recognized as DE in *Eichhornia* were also DE in *L. multiflorum*, including a gibberellin-regulated protein, oligopeptide transporter 7-like, exocyst complex component, cellulose synthase-like protein d4-like, auxin-responsive protein, and a myb family transcription factor family protein. This suggests that there may be similar genes involved in floral organ height and length across the angiosperms because the taxa are quite distantly related, with *Eichhornia* being a monocot and *L. multiflorum* being a dicot. Landis et al. (2015) studied two species of *Saltugilia* (V. E. Grant) L. A. Johnson (Polemoniaceae), one with small corollas and one with large corollas. These authors recognized over 700 DE genes comparing mature flowers of the two species, with many DE genes involved in cellular functions.

In a recent study of the transcriptomes of flowers of heterostylous morphs, Nowak et al. (2015) compared two species of *Primula, P. veris* and *P. vulgaris*. These authors identified ca. 650 genes as DE in each of the two species throughout development, although their FDR-adjusted *P*-value threshold was 0.05, not 0.1, which was employed in the present study. Additionally, these authors utilized floral tissue from only one stage of development, immature floral buds 3 to 5 days prior to anthesis. Comparing the two species, Nowak et al. (2015) found 113 genes that were DE in both, with more overexpressed in the SS morph than in the LS morph. This pattern of DE is similar to the situation in the gynoecium of *L. multiflorum*. The study of *Primula* focused, in part, on the identification of the *S*-locus, the supergene controlling the heterostylous condition. The genes identified as being linked to the *S*-locus in *P. veris*, including a GLOBOSA homolog expressed only in the SS morph, were not DE in the present study, and it should be noted that while the *S*-locus appears to strongly influence heterostyly in *Primula* (Fornoni and Domínguez, 2015; Nowak et al., 2015) its presence has yet to be identified in *Lithospermum* and other species of Boraginaceae. Therefore, the *S*-locus may control the development of the breeding system in *Primula*, but other genes and molecular interactions may govern the development of heterostyly in *Lithospermum* and its relatives. As further genomic information becomes available, it will be possible to more carefully and critically examine differences among heterostylous species.

Some of the genes that have previously been identified as involved in flower size [reviewed in Krizek and Anderson (2013)] were also DE between morphs of *L. multiflorum*. During early development of the gynoecium, cytochrome p450 93a1-like, which encourages increase in flower size (Anastasiou et al., 2007), was upregulated in the LS morph, and this was also the case for two auxin-response proteins and one auxin-induced protein. These types of genes have been demonstrated to increase floral organ size in other plants (Hu et al., 2003; Varaud et al., 2011). During late development, growth-regulating factors were upregulated in the corolla and androecium of the LS morph, and mutations in these genes have resulted in smaller petals (Kim et al., 2003). This is notable because the flowers of the LS morph of most heterostylous species, including *L. multiflorum* (Cohen et al., 2012), are smaller than those of the SS morph (Cohen, 2010).

In the corolla and androecium of the SS morph and in the gynoecium of the LS morph, the transcription factor tcp14-like is upregulated (Table 3). TCP transcription factors are known to regulate growth either through promotion or repression of growth (Martín-Trillo and Cubas, 2010). Given the pattern of expression of this TCP transcription factor, it is hypothesized that in *L. multiflorum* the putative transcription factor tcp14-like is involved in promoting growth, which would result in the larger corollas of the SS morph and the longer styles of the LS morph. Additionally, various genes involved in responding to auxin are DE, with most of these genes upregulated in the larger organs of the corolla and androecium of the SS morph and of the gynoecium of the LS morph. In *P. veris*, Huu et al. (2016) identified a gene, *CYP734A50*, that is a putative brassinosteroid-degrading enzyme that regulates style length. In *L. multflorum*, six genes, including brassinosteroid-regulated protein bru1, are involved in the brassinosteroid biosynthetic pathway. These genes are more highly expressed in the corolla and androecium of the LS morph and the gynoecium of the SS morph. This pattern is the opposite of that for auxin suggesting that this hormone may be involved in the development of smaller flowers and floral organs.

Given the distinct differences in herkogamy between floral morphs of heterostylous species and the importance of herkogamy in influencing pollination biology, genes involved in controlling floral organ height and length should be considered speciation genes (Rieseberg and Blackman, 2010). Mutations in these genes, such as auxin response factors (Hu et al., 2003; Varaud et al., 2011), may result in an allogamous species becoming autogamous or requiring a different pollinator to effectively transfer pollen between the anthers and stigmas of flowers that bear organs at different heights. As the function of the DE genes in the present study is further elucidated, particularly for genes also DE in flowers of other species, the importance of these genes on speciation will become evident, which will be aided by additional floral transcriptomes of species exhibiting variation in herkogamy.

### Identification of DE genes involved in response to stress

While the number of DE genes, between morphs, involved in response to stress could be due to the collection of plants from wild populations, it seems likely that given the biological replicates and the consistency among them as well as similar climate and weather patterns among collections and between years, the variation in stress response between morphs has a biological basis. The two morphs may have different genes expressed in response to stress or one morph may have a stronger response to stress than the other. Some of the DE genes involved in stress response are influenced by hormones known to be involved in this type of process, including ethylene and abscisic acid, and these genes include various ethylene-responsive transcription factors, abscisic acid receptor pyr1-like, and others (Thirugnanasambantham et al., 2015; Wang et al., 2015) (Table 7). Future research can investigate variation in hormones or hormone response between morphs, which may influence the observed morph-specific responses to stress.

Ralston (1993) provides evidence that the two morphs of *L. multiflorum* can respond differently to herbivory. She found that following herbivory individuals with longer styles increased seed production. Additionally, Ralston determined that, compared to the LS morph, the SS morph had significantly greater herbivory and seed production following herbivory. In a study on the heterostylous species *Gelsemium sempervirens* (L.) J. St.-Hil. (Gelsemiaceae), Leege and Wolfe (2002) provide evidence that floral herbivory is morph-specific. In *G. sempervirens*, the floral organs toward the apex of the floral tube receive a greater amount of herbivory, resulting in the styles of the LS morph and the stamens of the SS morph being more damaged than these same organs in the flowers of the other morph. These differences in herbivory may result in distinct responses to stress, which were observed in the present study. The different stress responses may be similar to contrasting SI responses between morphs.

## Future directions

Comparative transcriptomics provides a powerful tool for the identification of genes involved in heterostyly and the heights and lengths of floral organs, and this methodology allows for a variety of additional studies to be undertaken in *L. multiflorum* and other taxa. For example, in *L. multiflorum* it is now possible to examine patterns of variants of genes, including alternative splicing, not only genes that are DE between morphs. Additionally, investigating the patterns of DE genes throughout development of the flowers of the same morph can provide further insight into the molecular underpinnings of heterostyly within this species. An exciting area of research involves comparisons with other heterostylous species within the genus and family and across angiosperms as data collected can help identify whether a suite of genes involved in heterostyly is present in all heterostylous species [or all heterostylous species from a particular clade (e.g., Lamiidae)] or if new genes are recruited during each origin of heterostyly.

## Author contributions

JC planned, designed, and implemented the experiment. He was involved with collecting plants, laboratory research, data analysis and interpretation, and writing and editing the manuscript.

### Conflict of interest statement

The author declares that the research was conducted in the absence of any commercial or financial relationships that could be construed as a potential conflict of interest.

## Supplementary material

Table S1 **Morph, developmental stage, floral organs, and sequencing and mapping statistics on floral transcriptomes, C, corolla; A, androecium; G, gynoecium; PE, paired-end reds; SR, single reads**.

Table S2 **Results of Pearson correlation test for all analyzed floral transcriptomes of ***Lithospermum multiflorum*****. Numbers above diagonal are for isoforms, and numbers below diagonal are for components (putative genes).
